# Inhibition of Rac1 attenuates radiation-induced lung injury while suppresses lung tumor in mice

**DOI:** 10.1038/s41420-021-00791-8

**Published:** 2022-01-14

**Authors:** Ni An, Zhenjie Li, Xiaodi Yan, Hainan Zhao, Yajie Yang, Ruling Liu, Yanyong Yang, Fu Gao, Bailong Li, Hu Liu, Hongbin Yuan

**Affiliations:** 1 Department of Anesthesiology, Changzheng Hospital, Naval Medical University, Shanghai, China; 2 Anesthesiology Department, The 201th Hospital of the Chinese People’s Liberation Army, Liaoyang City, Shenyang province China; 3 Department of Radiation Medicine, Faculty of Naval Medicine, Naval Medical University, Shanghai, China; 4 Department of Radiation Oncology, Changhai Hospital Affiliated to the Naval Medical University, Shanghai, China; 5 College of Basic Medicine, Naval Medical University, Shanghai, China

**Keywords:** Apoptosis, Lung cancer

## Abstract

The lung is one of the most sensitive tissues to ionizing radiation, thus, radiation-induced lung injury (RILI) stays a key dose-limiting factor of thoracic radiotherapy. However, there is still little progress in the effective treatment of RILI. Ras-related C3 botulinum toxin substrate1, Rac1, is a small guanosine triphosphatases involved in oxidative stress and apoptosis. Thus, Rac1 may be an important molecule that mediates radiation damage, inhibition of which may produce a protective effect on RILI. By establishing a mouse model of radiation-induced lung injury and orthotopic lung tumor-bearing mouse model, we detected the role of Rac1 inhibition in the protection of RILI and suppression of lung tumor. The results showed that ionizing radiation induces the nuclear translocation of Rac1, the latter then promotes nuclear translocation of P53 and prolongs the residence time of p53 in the nucleus, thereby promoting the transcription of Trp53inp1 which mediates p53-dependent apoptosis. Inhibition of Rac1 significantly reduce the apoptosis of normal lung epithelial cells, thereby effectively alleviating RILI. On the other hand, inhibition of Rac1 could also significantly inhibit the growth of lung tumor, increase the radiation sensitivity of tumor cells. These differential effects of Rac1 inhibition were related to the mutation and overexpression of Rac1 in tumor cells.

## Introduction

The lung is one of the most sensitive tissues to ionizing radiation, thus, radiation-induced lung injury (RILI) stays a key dose-limiting factor of thoracic radiotherapy and is an inevitable companion of thoracic radiotherapy [1]. However, there is still little progress in the safe and effective treatment of RILI. Ideally, radio-protectors should have minimal side effects, and above all, do not exert protective effects on tumor cells. Therefore, seeking such a radio-protective agent or molecular target that can mediate such a bidirectional effect has become the key to improving tumor radiotherapy.

According to previous studies, Ras-related C3 botulinum toxin substrate1, Rac1, is small guanosine triphosphatases (GTPases) involved in oxidative stress [2–4], DNA damage [5, 6], and epithelial–mesenchymal transition (EMT) [7, 8], which are all important mechanisms of radiation-induced tissue damage. Thus, Rac1 may be an important molecule that mediates radiation damage, inhibition of which may produce a protective effect on RILI. In addition, Rac1 is over-expressed or mutated in various tumors [9]. The gain-of-function (GOF) mutation of Rac1 can lead to cancer-related phenotypes [10]. Rac1 not only participates in the formation, progression, invasiveness, and angiogenesis of tumors, but also mediates tumor cell resistance to radiotherapy, chemotherapy, and immunotherapy [11]. Inhibition of Rac1 can inhibit tumor growth and restore the sensitivity of patients to cancer therapy [11]. Therefore, inhibition of Rac1 has the potential of protecting normal tissues from radiation-induced injury, and at the same time, inhibiting tumor growth and sensitizing tumor to radiation therapy, which makes it a promising ideal molecular target for the protection and treatment of radiation-induced lung injury caused by clinical radiotherapy.

In this study, the role of Rac1 in a mice model of RILI was investigated, and the influence of regulating Rac1 expression on the development of lung cancer was explored.

## Results

### Radiation-induced lung injury was alleviated by Rac1 inhibition

**Fig. 1:**
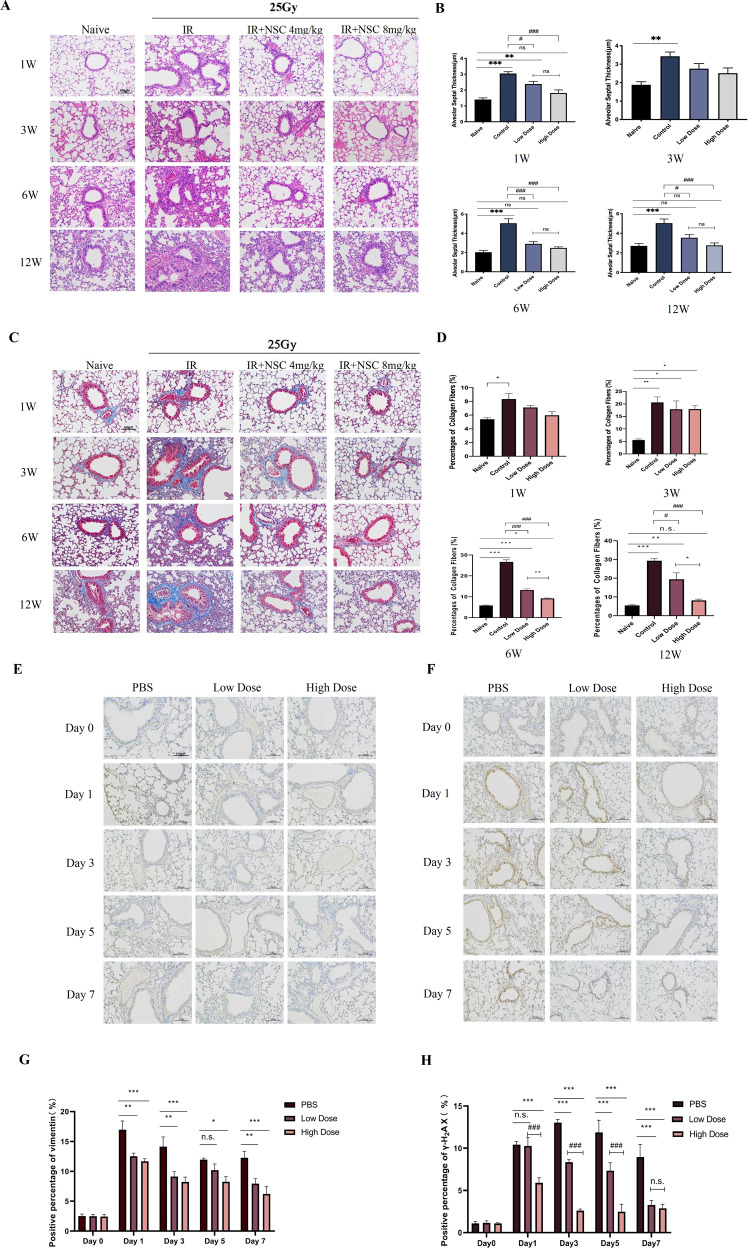
Radiation-induced lung injury was alleviated by Rac1 inhibition. C57BL/6 were randomly divided into four groups which were Naive (no IR + PBS), IR (IR + PBS), Low-Dose (IR + 4 mg/kg NSC23766) and High-Dose (IR + 8 mg/kg NSC23766) (*N* = 20). Rac1 inhibition was achieved by intraperitoneal injection of NSC23766, a specific Rac1 inhibitor. Intraperitoneal injection of PBS or NSC23766 was given once a day for a consecutive three days. 2 h after the 3^rd^ injection, all mice except those of the Naive group were anesthetized and immobilized in a radiation-specific box for 25 Gy of local lung irradiation (IR). On the 1^st^, 3^rd^, 6^th^, and 12^th^ week after IR, 5 mice from each group were sacrificed and lung tissues were collected. **A**, **B** Hematoxylin and eosin (H&E) staining and **C**, **D** Masson staining was conducted for the evaluation of pulmonary inflammation and fibrosis, respectively. **E**–**H** C57BL/6 mice were randomly divided into three groups, namely the PBS group (IR + PBS), the Low-Dose group (IR + 4 mg/kg NSC23766), and the High-Dose group (IR + 8 mg/kg NSC23766) (*N* = 15). Before radiation treatment (day 0) and on the 1^st^, 3^rd^, 5^th^, and 7^th^ days after IR, lung tissues from 3 mice of each group were collected for immunohistochemistry analysis of vimentin and γ-H_2_AX, the important indicator of pulmonary epithelial–mesenchymal transformation (EMT) and DNA damage respectively. Image analysis was conducted using Image J software. ns represented that there was no statistically significant difference between the two groups. *, **, and *** represented *P* < 0.05, 0.01, and 0.001 between the corresponding groups, respectively. ^#^, ^##^, and ^###^ represented *P* < 0.05, 0.01, and 0.001 between the corresponding groups, respectively.

Compared with the Naive group, the alveolar septum of the IR group was significantly thickened and the alveolar infiltrated with inflammatory cells (Fig. 1A, B), the collagen fibers in the lung of the IR group was increased significantly from the first week and the proportion of collagen fibers gradually increased from the 3^rd^ week to the 12^th^ week (Fig. 1C, D), indicating that pulmonary inflammation and fibrosis was induced by IR. In contrast, intraperitoneal injection of NSC23766 could significantly alleviate RILI both in the Low-dose (4 mg/kg) and the High-dose (8 mg/kg) groups (Fig. 1A–D). Compared with the IR group, the upregulation of Vimentin, an important indicator of epithelial–mesenchymal transition (EMT), induced by radiation, was also inhibited by NSC23766 treatment (Fig. 1E, G). Besides, γ-H_2_AX (an indicator for DNA damage) was upregulated from day 1 to day 7, demonstrating a simultaneous occurrence of DNA damage with pulmonary inflammation. NSC23766 treatment significantly inhibited the upregulation of γ-H_2_AX from day 3 to day 7 after irradiation (Fig. 1F, H). These results supported that inhibition of Rac1 could produce a protective effect on the lung tissue against RILI of mice.

### Radiation-induced injury of mouse lung epithelial cell line was alleviated by Rac1 Inhibition/Knockdown

**Fig. 2:**
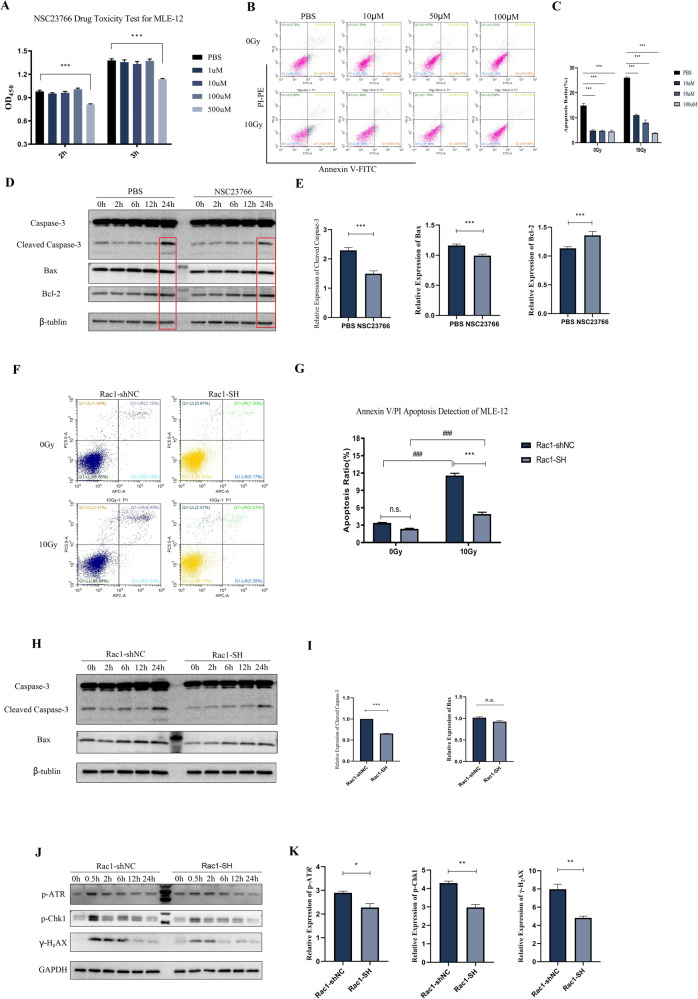
Radiation-induced apoptosis and DNA damage of MLE-12 were reduced by knockdown and inhibition of Rac1. **A** CCK-8 drug toxicity test of NSC23766 for MLE-12. Cells were treated with PBS or different doses of NSC23766 (1, 10, 100, and 500 μM) for 24 h and then tested with CCK-8. **B**, **C** MLE-12 cells were pretreated with PBS or different doses of NSC23766 (10, 50, and 100 μM) for 2 h before 0 Gy (no radiation) or 10 Gy of radiation. The apoptosis ratio was detected by the Annexin V/PI method with a flow cytometry 24 h after radiation. **D**, **E** Cells were pretreated with PBS or 100 μM NSC23766 for 2 h and collected at designed time points after 10 Gy of radiation for WB detection of the expression of Caspase-3 and Bax. Rac1 knockdown (Rac1-SH) and control (Rac1-shNC) MLE-12 cell lines were constructed using Lentivirus vector. Cells were collected before (0 Gy) and at designed time points after 10 Gy of radiation. **F**, **G** Apoptosis ratio was detected using Annexin V/PI method by a flow cytometer. **H**, **I** Apoptosis-related proteins, Bax and Caspase-3 were detected by WB analysis. **J**, **K** The expression of DNA damage-repair related proteins, p-ATR, p-Chk1, and γ-H2AX were also tested by WB analysis. *** represented *P* < 0.001 between the two groups. The error value was expressed as mean ± SEM. The experiment was repeated three times.

MLE-12 was used for the investigation of the effects of Rac1 inhibition in vitro. Cell apoptosis was significantly induced (Fig. 2B, C), and the expression of apoptosis-related proteins, Cleaved Caspase-3 and Bax, was significantly upregulated by 10 Gy of radiation (Fig. 2D, E). The expression levels of p-ATR, p-CHK1, and γ-H_2_AX were increased from 0.5 h after irradiation and gradually recovered until 24 h later (Fig. 2J, K). 100 μM NSC23766 pre-treatment (dose determined by CCK-8 Test in Fig. 2A) could reduce the apoptosis ratio (Fig. 2B, C) and decrease the upregulation of Cleaved Caspase-3 and Bax at 24 h after radiation (Fig. 2D, E).

Further, Rac1 knockdown (Rac1-SH) and control (Rac1-shNC) MLE-12 cell lines were constructed. The knockdown efficiency was verified by WB, qPCR, and fluorence intensity detection (Supplemental Material, S1). The apoptosis ratio of Rac1-SH MLE-12 was significantly lower than that of the control group at 24 h after 10 Gy of radiation (Fig. 2F, G), in accordance with this, the expression level of Cleaved Caspase-3 was p-ATR, p-CHK1 and γ-H_2_AX were all significantly lower than those of Rac1-shNC MLE-12 after 10 Gy of radiation (Fig. 2H–K). Collectively, our results indicated that radiation-induced apoptosis and DNA damage of MLE-12 were alleviated by Rac1 inhibition/knockdown.

### RNA-Seq analysis of differentially expressed genes caused by Rac1 knockdown and identification of Trp53inp1 as the critical downstream target

**Fig. 3:**
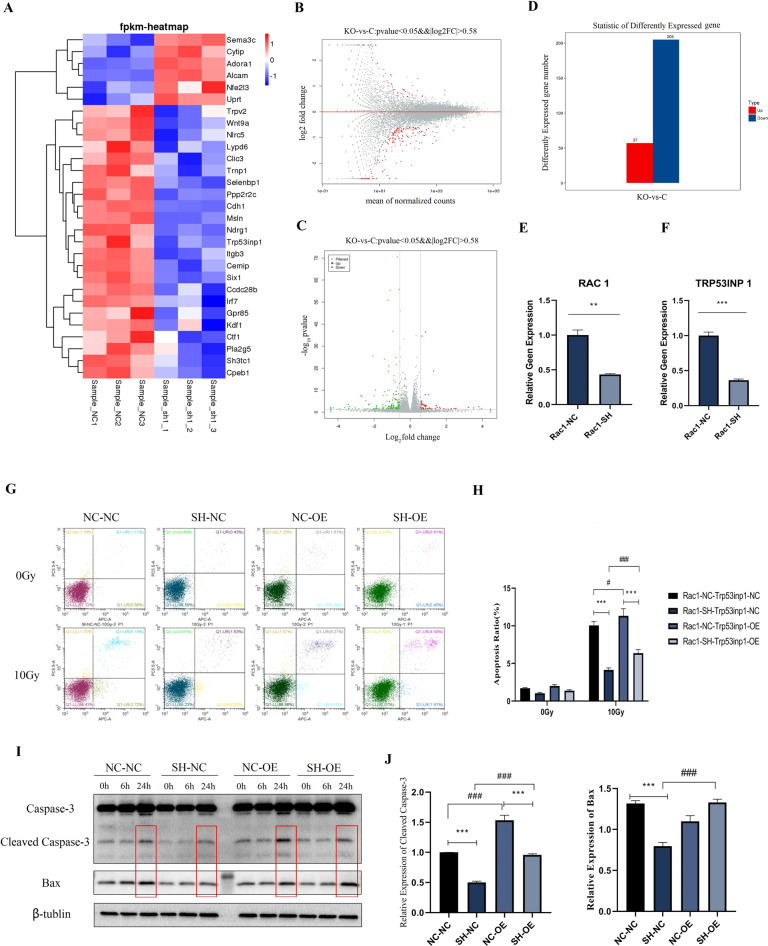
Identification of Trp53inp1 as the critical downstream target of Rac1 by RNA-Seq analysis. RNA-seq and qPCR analysis of differential expression genes between Rac1 knockdown (Rac1-SH) and control (Rac1-NC) MLE-12. **A** Cluster analysis results of differential expression genes. **B**–**D** MA diagram, Volcano map, and histogram of differential expression genes. Red column represents the genes upregulated by Rac1 knockdown, and blue represents the genes downregulated. *P* < 0.05, |log 2FC| > 0.58. A total of 262 differential genes were caused by Rac1 knockdown, of which 205 genes were downregulated and 57 genes were upregulated. **E**, **F** qPCR analysis of relative gene expression level of Rac1 and Trp53inp1 between Rac1-SH and Rac1-NC MLE-12. Trp53inp1 overexpression (OE) and negative control (NC) MLE-12 cell lines were constructed on the basis of Rac1-NC and Rac1-SH MLE-12 cells, yielding a total of 4 kinds of genetically modified MLE-12 cells, which were Rac1-NC+ Trp53inp1-NC (represented as NC-NC), Rac1-SH+ Trp53inp1-NC (represented as SH-NC), Rac1-NC+ Trp53inp1-OE (represented as NC-OE) and Rac1-SH-Trp53inp1-OE (represented as SH-OE). All 4 kinds of cells were collected before (0 Gy) and at designed time points after 10 Gy of irradiation. **G**, **H** Apoptosis ratio detected by a flow cytometer. **I**, **J** Apoptosis-related proteins, Bax and Caspase-3 were detected by WB analysis. ** and *** represented *P* < 0.01 and 0.001 between the corresponding groups, respectively. The error value was expressed as mean ± SEM. Experiments were repeated three times.

To further investigate the downstream target of Rac1 knockdown, RNA-seq analysis was performed. Cell lysates from Rac1-SH MLE-12 and Rac1-shNC (control) MLE-12 were collected (N = 3) for RNA-seq analysis. Quality control results proved that the within-group difference of each group was small and the correlation between the two groups was good (Supplemental Material, S2A–E). Differential genes screening between groups was based on the significant level of *P* < 0.05 and fold change of |log 2FC|>0.58. A total of 262 differential genes were caused by Rac1 knockdown, of which 205 genes were downregulated and 57 upregulated (Fig. 3A–D). Gene ontology (GO) analysis and KEGG pathway analysis showed that the differential genes caused by Rac1 knockdown are mostly involved in cellular processes of transport and catabolism, and cell growth and death, and are related to human diseases including infectious disease and cancers, and may influence genetic information processes including replication and repair (Supplemental Material, S2F, G).

According to the molecular biological functions of differential genes and relative literature research, 14 genes of interest were further analyzed by qPCR. Compared with the control group, the relative expression level of Rac1 in the Rac1-SH MLE-12 was significantly lower (*P* < 0.01) (Fig. 3E), proving that the Rac1 knockdown cell line was successfully constructed, 8 of 14 genes were significantly downregulated or upregulated and changes were consistent with results from the RNA-seq analysis (Supplemental Material, S3A–G and Fig. 3F), 6 of 14 genes were not significantly changed (*P* > 0.05) while their trends of upregulation or downregulation were consistent with results from the RNA-seq analysis (Supplemental Material, S3H–M). qPCR results further validated the reliability of RNA-seq. Among these genes, TRP53INP1 was of most interest, which was significantly downregulated by Rac1 knockdown (*P* < 0.001) (Fig. 3F).

Human tumor protein p53-induced nuclear protein 1 (TP53INP1), also known as TEAP/SIP/p53DINP1, is a p53 target gene that is mainly expressed in the nucleus [12] and mediates p53-dependent apoptosis [13]. The same gene of mice is named Trp53inp1. According to previous research [12], various cellular stress stimuli including γ-radiation could induce the expression of TP53INP1 which further stimulates the transcriptional activation of p53 target gene promoters, such as Bax, which supports that Trp53inp1 is likely to play a role in cell damage caused by radiation.

The Trp53inp1 overexpression (OE) and negative control (NC) MLE-12 cell lines were constructed on the basis of Rac1-NC and Rac1-SH MLE-12 cells, yielding a total of 4 kinds of genetically modified MLE-12 cells, which were Rac1-NC+ Trp53inp1-NC (represented as NC-NC), Rac1-SH+ Trp53inp1-NC (SH-NC), Rac1-NC+ Trp53inp1-OE (NC-OE) and Rac1-SH+ Trp53inp1-OE (SH-OE). The apoptosis ratio of the four groups was similar before IR treatment, namely 0 Gy, and were all significantly increased 24 h after 10 Gy of radiation (Fig. 3G, H). Compared with the SH-NC group, the apoptosis ratio of Rac1 knockdown combined with Trp53inp1 overexpression (SH-OE) was significantly higher (*P* < 0.001) but still lower than the NC-NC group (Fig. 3G, H). Next, the expression of apoptosis-related proteins was investigated. 24 h after IR, the expression levels of Cleaved Caspase-3 and Bax in the four groups were increased. However, compared with the SH-NC group, the expression levels of Cleaved Caspase-3 and Bax in the SH-OE group were also significantly higher (Fig. 3I–J). The above results indicated that overexpression of Trp53inp1 could partially reverse the apoptosis-reducing effect of Rac1 knockdown on MLE-12 after radiation.

### Binding of Rac1 with p53 was induced by radiation, which further mediating the transcription of Trp53inp1

**Fig. 4:**
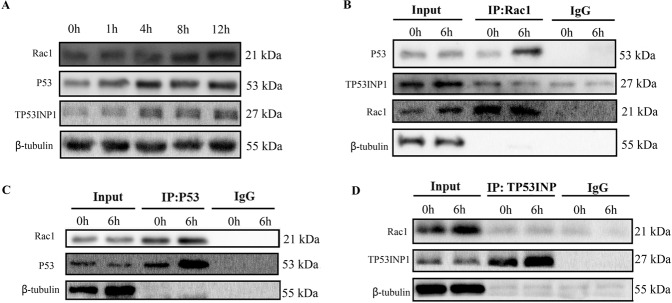
Binding of Rac1 with P53 was induced by radiation. **A** MLE-12 cells were radiated with 10 Gy, and the expression of Rac1, P53, and Trp53inp1 were detected by Western blotting at a designed time (0, 1, 4, 8, 12 h after radiation). **B** At 6 h after radiation, immunoprecipitation was conducted to detect the binding of Rac1 with p53 and Trp53inp1, in which Rac1 was precipitated. **C**, **D** At 6 h after radiation, p53 (**C**) and Trp53inp1 (**D**) was precipitated, respectively, and then the expression of Rac1 was detected.

The above results proved that Trp53inp1 was the key downstream molecule that mediated the radiation-induced damage effects of Rac1. Combining with previous studies, these indicated that there existed a regulatory relationship between Rac1, p53, and TP53INP1, and this regulatory relationship affects the radiation sensitivity of cells. Therefore, the relationship between Rac1, p53, and TP53INP1 was studied. The expression of Rac1, p53, and TP53INP1 was all time-dependently increased after irradiation (Fig. 4A). Co-immunoprecipitation showed that the proteins precipitated by the antibody of Rac1 contained p53 but did not contain Trp53inp1, and that the content of p53 was significantly increased 6 h after irradiation (Fig. 4B). Further, co-immunoprecipitation using antibodies against p53 and TP53INP1 showed the Rac1 protein could be precipitated by the antibody against p53 (Fig. 4C) while not that against TP53INP1 (Fig. 4D). The above results indicated that Rac1 is mainly bound to p53 instead of TP53INP1 after irradiation. Thus, the increase in the expression of Trp53inp1 mainly depends on the binding of Rac1 to p53 to promote the transcription of Trp53inp1.

### Radiation induces nuclear translocation of Rac1 and subsequently prolonged the residence time of p53 in the nucleus

**Fig. 5:**
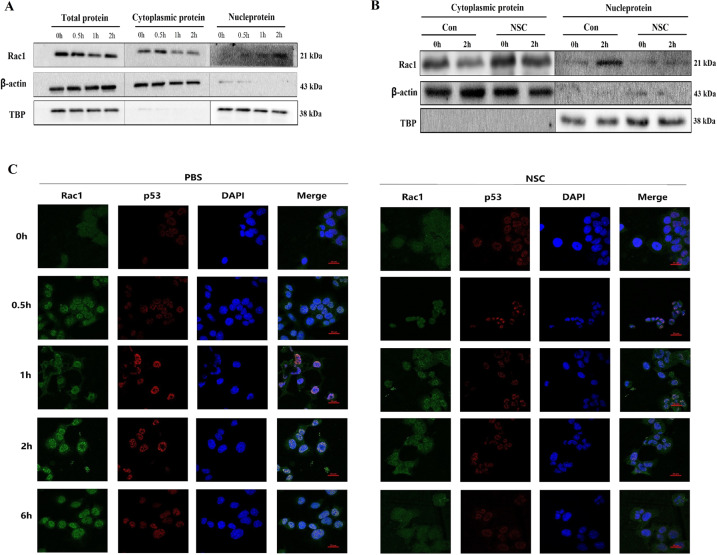
Nuclear translocation of Rac1 was induced by radiation, which subsequently prolonged the residence time of p53 in the nucleus. MLE-12 cells were pretreated with PBS or NSC23766 (NSC, 100 μM) for 2 h and then went through 10 Gy of radiation. **A** At 0–2 h after radiation, nucleoprotein, and cytoplasmic protein were separated for detection of the subcellular distribution of Rac1 and p53. **B** At 2 h after radiation, the translocation of Rac1 and p53 were detected with or without NSC23766 (NSC, 100 μM) pre-treatment. **C** Cells were collected from 0 to 6 h after radiation for immunofluorescence analysis of the translocation of Rac1 and p53. Green and Red fluorescence represented Rac1 and p53, respectively.

Our previous results showed that the radiation protective effect of Rac1 inhibition/knockdown on MLE-12 was partially mediated by downregulating Trp53inp1. According to the previous study [14], when cells faced damage caused by ionizing radiation, p53 translocated from the cytoplasm into the nucleus and initiated the transcription of Trp53inp1, thereby inducing cell cycle arrest and enhancing p53-mediating apoptosis. Therefore, inhibition of Rac1 may affect the reaction of p53 to radiation stimulation, thereby further downregulating the expression of Trp53inp1. Thus, the cytoplasmic protein and nucleoprotein of MLE-12 were separated for detection of subcellular localization of Rac1 before (0 h) and after 10 Gy of radiation. The expression levels of Rac1 in the nucleoprotein were increased from 30 min after radiation and peaked at 2 h after (Fig. 5A). However, the translocation of Rac1 to the nucleus was inhibited by NSC23766 (Fig. 5B).

Next, the localization of Rac1 and p53 before and after 10 Gy of radiation was investigated by confocal microscopy. Results showed that the translocation of Rac1 and p53 from the cytoplasm to the nucleus started for 30 min, peaked at 2 h, and subsided at 6 h after radiation (Fig. 5C). Compared with the PBS group, the nuclear translocation of Rac1 was reduced and subsided earlier (from 2 h after radiation) in the NSC group (treated with 100 μM NSC23766), and the residence time of p53 in the nucleus and its expression level was significantly reduced (Fig. 5C). These suggested that ionizing radiation induces the co-localization of P53 and Rac1 in the nucleus and prolongs the residence time of p53 in the nucleus, thereby promoting p53-dependent apoptosis. Rac1 inhibition could inhibit this nuclear translocation of Rac1, thereby reducing cell apoptosis by shortening the residence time of p53 in the nucleus.

### Rac1 Inhibition/Knockdown attenuates radiation-induced lung injury while sensitizes lung tumor to radiotherapy

**Fig. 6:**
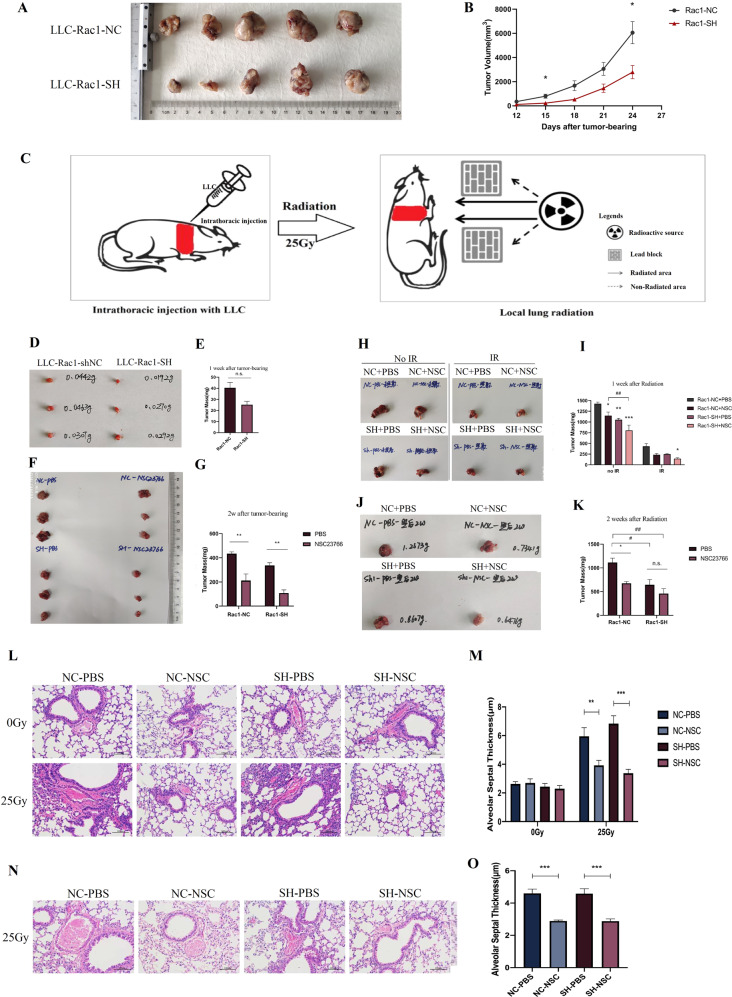
In vivo verification of the differential effects of Rac1 inhibition/knockdown, inhibiting lung tumor while protecting normal lung tissue. **A**, **B** BALB/cJGpt-Foxn1^nu^/Gpt mice were randomly divided into two groups, namely the control group (LLC-Rac1-NC) and the Rac1 knockdown group (LLC-Rac1-SH). After ear labeling, mice were subcutaneously injected with 1 × 10^6^ of corresponding LLC cells on the right hind leg. The control group was injected with Rac1-NC LLC and the Rac1 knockdown group with Rac1-SH LLC. The size of each mouse’s tumor was measured with a vernier caliper on the 12^th^, 15^th^, 18^th^, 21^st^, and 24^th^ days after tumor-bearing. Tumor volume (mm^3^) was calculated as the longest diameter (mm) × the shortest diameter (mm)^2^. **C** Schematic diagram of orthotopic lung tumor-bearing and local lung radiotherapy. **D**–**K** Orthotopic lung tumor-bearing mouse model. The specific study design was written in detail in the part of Materials and Methods. The tumor volumes of Rac1 knockdown group on the 15^th^ and 24^th^ days after tumor-bearing were significantly smaller than those of the control group (*P* < 0.05). **L**–**O** H&E staining of non-tumor-bearing contralateral lung tissues. NC-PBS, NC-NSC, SH-PBS, and SH-NSC represented tumor-bearing with Rac1-NC or Rac1-SH LLCs combined with an intraperitoneal injection of PBS or NSC23766 (3 mg/kg, on alternate days for a week). **M**, **O** were the statistical analysis of the thickness of each group’s alveolar septum thickness on 1 and 2 weeks after local lung irradiation. *, **, and *** represented *P* < 0.05, *P* < 0.01, and *P* < 0.001 between the corresponding groups, respectively. The error value was expressed as mean ± SEM. *N* = 3.

After confirming the protective effects of Rac1 inhibition on RILI, the effects of Rac1 inhibition on lung tumor was further investigated. First, a subcutaneous tumor-bearing nude mice model was constructed with Rac1-SH or Rac1-NC LLC. The final statistics were 7 for each group. The tumor volumes of the Rac1 knockdown group on the 15th and 24th days after tumor-bearing were significantly smaller than those of the control group (*P* < 0.05) (Fig. 6A, B), indicating that the growth of mouse lung cancer cells was significantly inhibited by Rac1 knockdown.

Next, an orthotopic lung tumor-bearing mouse model was designed. Figure 6C was the illustration of orthotopic lung tumor-bearing and local lung radiation. There was a significant difference in tumor mass between the two groups at basal condition (*P* = 0.028) (Fig. 6D, E). After 1 week of PBS or NSC23766 (NSC) administration in the 2nd week, within the same main group (NC or SH group), the tumor mass of the NSC subgroup was significantly smaller than that of the PBS subgroup (both *P* < 0.01) (Fig. 6F, G). 3^rd^ week after tumor-bearing that was 1 week after radiation, each tumor (NC + PBS, NC + NSC, SH + PBS, SH + NSC group, respectively) mass under IR was all significantly smaller than that of their corresponding no IR group (all *P*-values < 0.001) (Fig. 6H, I). By the end of the 4th week after tumor-bearing, the tumor mass of the NC + NSC and SH + PBS group were significantly smaller than that of NC + PBS group (*P* < 0.05) (Fig. 6J, K), the difference between those of the SH + NSC group and the NC + PBS group was even more significant (*P* < 0.01) (Fig. 6J, K).

H&E staining of the contralateral non-tumor-bearing lungs was also performed to verify the protective effect of Rac1 inhibition on RILI, and results demonstrated that 1 week after radiation, the thickness of alveolar septum in the NC + NSC group and the SH + NSC group were significantly smaller than those of the NC + PBS group and the SH + PBS group, respectively (*P* < 0.01 and *P* < 0.001, respectively) (Fig. 6L, M), similar results were seen on 2 weeks after radiation (both *P* < 0.001) (Fig. 6N, O).

These results showed that the growth of LLC cells was significantly inhibited by Rac1 knockdown and that the combination of Rac1 knockdown and inhibition had a stronger inhibitory effect on tumor growth, while protecting RILI.

### The mutation of Rac1 in lung cancer cells was responsible for the differential reactions in post-radiation ROS level, proliferation ability, and apoptosis ratio between lung cancer cells and normal lung epithelial cells

**Fig. 7:**
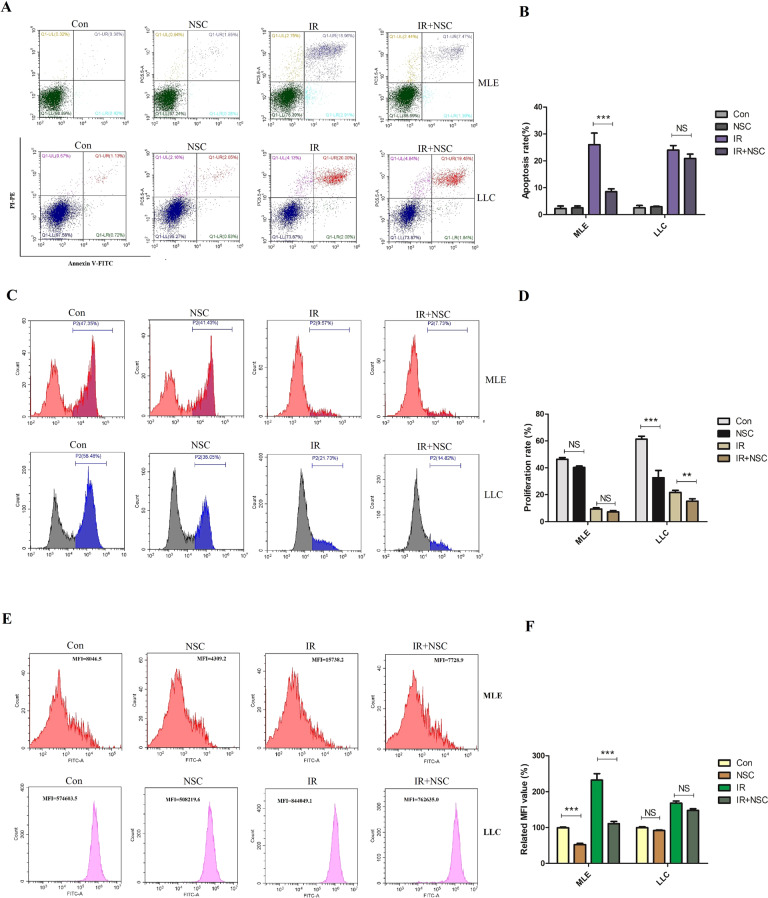
The effects of Rac1 inhibition on the apoptosis, proliferation and ROS levels of MLE and LLC. MLE and LLC were pretreated with PBS or NSC23766 (NSC, 100 μM) for 2 h and then went through 10 Gy of radiation. 24 h later, the apoptosis ratio (**A**) and potential of proliferation (**C**) were detected by Annexin V/PI method and Edu labeling with flow cytometry, respectively. At 6 h after radiation, the ROS content (**E**) was detected by ROS probe with flow cytometry. Panels **B**, **D**, and **F** were the quantification for **A**, **C**, and **E**, respectively. *** represented *P* < 0.01 and 0.001 between the corresponding groups, respectively. The error value was expressed as mean ± SEM. Experiments were repeated three times.

By now, we have found that inhibition of Rac1 could not only exert protective effects on RILI, but could also effectively inhibit the proliferation ability and increase the radiation sensitivity of lung tumor cells. The protein expression level and gene sequence of Rac1 in MLE-12 and LLC were analyzed. Compared with MLE-12, the expression level of Rac1 in LLC was significantly increased (Supplemental Material, S4A, B). Exon sequencing showed that there were two insertion segments in the RAC1 gene of LLC, compared with that of MLE-12, and since there existed overlap in the base calling of the insertion segments, there could exist multiple segments (Supplemental Material, S4C, D). Since the effects of Rac1 on cells mainly include three aspects, namely ROS induction, proliferation, and apoptosis regulation. Therefore, the effects of Rac1 inhibition on the post-radiation ROS production, proliferation ability and apoptosis ratio of MLE-12 and LLC were compared. Results showed that apoptosis of MLE-12 after radiation was significantly reduced by NSC23766, while that of LLC was not (*P* < 0.001, Fig. 7A, B). The proliferation ability of LLC was significantly inhibited by NSC23766 at basal condition (before radiation), and this inhibitory effect was further intensified after radiation (*P* < 0.001, Fig. 7C, D), while that of MLE-12 was little affected (*P* > 0.05, Fig. 7C, D), indicating that the proliferation of lung cancer cells was more dependent on Rac1. Besides, ROS probe was used to detect the contents of ROS in the two cells, results showed that the ROS content in LLC was significantly higher than that in MLE-12, while inhibition of ROS by NSC23766 was significant in MLE-12 other than LLC (*P* < 0.001, Fig. 7E, F).

## Discussion

This study mainly investigated the role of Rac1 inhibition in the alleviation of RILI and further researched its effect on radiotherapy of lung tumor in mice. Firstly, with the mouse model of RILI, the protective effect of Rac1 inhibition was verified. Next, MLE-12 cells were used to test the influence of Rac1 inhibition on radiosensitivity. Flow cytometry apoptosis detection and WB analysis showed that inhibition/knockdown of Rac1 could significantly reduce the apoptosis and DNA damage of MLE-12 after radiation, which were consistent with the results from animal experiments, supporting the radiation protective effects of Rac1 on normal lung tissue cells. Further, RNA-seq analysis was performed to further screen the downstream genes, and qPCR identified Trp53inp1 as the most significant target.

Our results showed that the expressions of Rac1 and Trp53inp1 were strongly correlated with each other, Trp53inp1 was significantly downregulated after Rac1 knockdown. However, Rac1 itself did not mediate the transcriptional function on Trp53inp1, its regulatory role on Trp53inp1 could be mediated by other molecules. Therefore, we focused on the relationship among Rac1, p53, and TP53INP1. Previous studies have shown that Rac1 could interact with p53 and play an important role in the proliferation and metastasis of various tumor cells [15], and that p53 is an important transcription factor for Trp53inp1 [13]. Co-immunoprecipitation showed that the binding of Rac1 with p53 was significantly promoted by irradiation, meanwhile, Rac1 did not bind with TP53INP1. These results not only validate our previous conjecture, but also coincide with previous studies. To our surprise, observing the binding of Rac1 with p53 through a confocal microscope also found that irradiation caused the nuclear translocation of Rac1, and after binding with p53 in the nucleus, it seemed to prolong the residence of p53 in the nucleus, which could eventually lead to the increase of apoptosis-related molecules in the cell. After Rac1 inhibition, the nuclear-translocation of Rac1 was inhibited and the “retention” effect on p53 was weakened. This phenomenon well explains the protective effect of Rac1 inhibition on RILI. As far as we know, this is the first study reporting the role of Rac1 nuclear-translocation on p53-mediated apoptosis. However, this effect of Rac1 on p53 was not studied in Rac1-knockout cells, because after Rac1 knockout, the binding of Rac1 with downstream molecules cannot be detected by co-immunoprecipitation. Therefore, the conclusion of this study is limited to inhibition of Rac1 activation, whether p53 would be regulated through other pathways after Rac1 knockout still needs further study.

We have now proved that Rac1 inhibition could effectively reduce RILI, however, discussing the prevention and treatment of RILI must consider the impact on tumor radiotherapy. To this end, nude mouse subcutaneous tumor-bearing model and orthotopic lung tumor-bearing model were constructed to further study the effects of Rac1 inhibition on tumors. It’s delighted to find that inhibition of Rac1 did not exert a protective effect on tumor cells against radiation, but also strengthened the tumor suppression effect. These differential effects of Rac1 inhibition are intriguing. The effects of Rac1 on cells mainly include three aspects, namely ROS induction, proliferation, and apoptosis regulation, which are also the most important mechanisms during radiation injury. Therefore, we further compared the proliferation, apoptosis, and ROS level of MLE-12 and LLC cells after irradiation. Results showed that Rac1 inhibition mainly reduced the post-radiation apoptosis and ROS production of MLE-12, with little influence on its proliferation ability. As for LLC, Rac1 inhibition significantly inhibited its proliferation ability before and after radiation, while affecting little on the apoptosis and ROS production of LLC after radiation. This well explains the differential effects of Rac1 inhibition. For normal lung epithelial cells, the proliferation cycle is longer, inhibition of Rac1 does not significantly affect its proliferation but can significantly reduce the post-radiation ROS production and apoptosis, which ultimately shows a protective effect. However, Rac1 is over-expressed in tumor cells. Many studies have shown that Rac1 is an important molecule that mediates tumor cell proliferation [9–11]. Therefore, Rac1 inhibition could significantly suppress tumor growth. On the other hand, our results also showed that inhibiting Rac1 has little effect on the apoptosis of LLC after radiation, without this protective effect seen in normal lung epithelial cells, Rac1 inhibition ultimately exert an anti-tumor effect. Rac1 inhibition produced these differential effects on normal cells and tumor cells, which could be related to the mutation of Rac1 in tumor cells. Many studies have shown that Rac1 is over-expressed and mutated mutations in various tumors, which is an important reason for the abnormal tumor proliferation and metastasis [9–11]. To confirm this, the protein expression and gene sequence of Rac1 in MLE and LLC were detected. Consistent with previous studies, the expression of Rac1 in LLC was significantly higher than that of MLE, and there were insertion mutations in the gene sequence of Rac1 in LLC. However, the exact function of this type of mutation was not investigated in this study. At present, the most common type of Rac1 mutation in tumor has been reported as the Rac1b mutation, which has been identified in melanoma and breast cancer [16]. Unfortunately, our sequencing results did not find Rac1b mutation in LLC (data not shown), which may be due to different tumor types. Anyway, our research still confirmed that the Rac1 in LLC was indeed mutated. Whether this kind of mutation is the cause of the differential effects of Rac1 still needs further investigation.

**Fig. 8:**
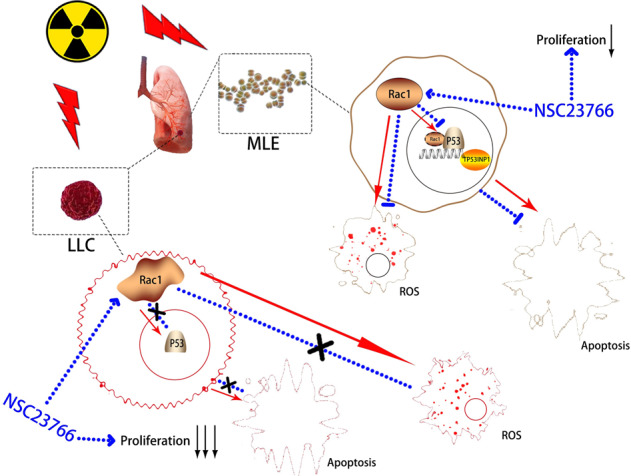
Summary of study. Ionizing radiation induces the nuclear translocation of Rac1, the latter then binds with p53 and prolongs the residence time of p53 in the nucleus, thereby promoting the transcription of Trp53inp1 which mediates p53-dependent apoptosis. Inhibition of Rac1 with NSC23766 could reduce the nuclear translocation of Rac1 induced by irradiation, thereby fasting the release of p53 from the nucleus, at last reducing the apoptosis. In addition, Rac1 inhibition could also significantly reduce ROS generation, but have no effect on the proliferation of MLE. In contrast, due to the overexpression and mutation of Rac1, inhibition of Rac1 does not reduce the apoptosis of tumor cells after radiation but can inhibit the proliferation ability of tumor cells significantly.

Collectively, the effects of Rac1 inhibition on normal lung epithelial cells were mainly acting as inhibition of post-radiation apoptosis and ROS production, while the proliferation ability was little affected, therefore, the overall effect presented as protection on RILI. As for LLC, Rac1 inhibition could significantly inhibit its proliferation ability before and after radiation, while affecting little on the apoptosis and ROS production of LLC after radiation, thus the overall effect presented as tumor suppression. These differential effects of Rac1 inhibition on normal and tumor cells could be due to the discrepancy in the basal expression level and gene sequence of Rac1 in different kinds of cells (Fig. 8).

In summary, our study supported that Rac1 inhibition could protect normal lung tissue from, and at the same time, sensitize lung tumors to radiation-induced damage, making it an important target for clinical radiation protection and tumor radiotherapy.

## Materials and methods

### Experimental animals

Wild-type C57BL/6 male mice at 6–8 weeks of age were purchased from the Experimental Animal Center of Naval Medical University. BALB/cJGpt-Foxn1^nu^/Gpt (nude) male mice at 6–8 weeks of age were purchased from GemPharmatech Co, Ltd. Mice were kept in the animal room of the Department of Radiation Medicine, Faculty of Naval Medicine, Naval Medical University, in a 12h–12h day–night rhythm with plenty of food and water. All animal operating met the requirements of the ethics committee of Naval Medical University.

### Radiation-induced lung injury mouse model

C57BL/6 mice were randomly divided into four groups, namely the naive group, the control group, the low-dose group, and the high-dose group, the former two groups were given an intraperitoneal injection of PBS and the latter two groups of 4 and 8 mg/kg NSC23766 (Selleck Corp., Ltd, Shanghai, China), respectively. After three days of injection, all groups except the naive group were treated with 25 Gy local lung radiation using the ^60^Co radiation source provided by the Radiation Center of Naval Medical University at a dose rate of 1 Gy/min. Mice were anesthetized with 1% Pelltobarbitalum Natricum and fixed in a radiation-specific box before radiation treatment. On the 1^st^, 3^rd^, 6^th^, and 12^th^ week after local lung radiation, three mice from each group were sacrificed by cervical dislocation and bilateral lungs were collected for H&E and Masson staining. On the 1st (day 1), 3rd (day 3), 5th (day 5), and 7th (day 7) days after local lung radiation, three mice from each group were sacrificed by cervical dislocation, and lungs were collected for immunohistochemistry analysis of vimentin, TGF-β and γ-H_2_AX.

### Subcutaneous tumor-bearing of nude mice

Twenty male BALB/cJGpt-Foxn1^nu^/Gpt mice were randomly divided into two groups, namely the control group and the Rac1 knockdown group (*N* = 10). After ear labeling, mice were subcutaneously injected with 1 × 10^6^ of corresponding LLC cells on the right hind leg. The control group was injected with Rac1-NC LLC and the Rac1 knockdown group with Rac1-SH LLC. The size of each mouse’s tumor was measured with a vernier caliper on the 12^th^, 15^th^, 18^th^, 21^st^, and 24^th^ days after tumor-bearing. Tumor volume (mm^3^) was calculated as the longest diameter (mm) × the shortest diameter (mm)^2^.

### Orthotopic lung tumor-bearing mouse model

70 male C57BL/6 were randomly divided into two main groups, the NC group, and the SH group (*N* = 35). Two kinds of LLC cells, namely the Rac1-NC LLC (control) and the Rac1-SH LLC (Rac1 knockdown), were digested with EDTA-containing trypsin and resuspended for cell counting. Each mouse was injected with 2 × 10^6^ of corresponding LLC cells suspended in a 50 μl mixture of 1:1 medium and matrigel, on the right lung through the incision below the right axillary. One week after tumor-bearing, 3 mice from each group were sacrificed by cervical dislocation and the tumor mass of each mouse was measured. From the 2nd week, each of the two main groups was further divided into two subgroups by different intraperitoneal injection of PBS or 3 mg/kg NSC23766 (NSC for short) on alternate days for a week, yielding a total of four groups, NC + PBS group, NC + NSC group, SH + PBS group and SH + NSC group (*N* = 15). After 1 week of intraperitoneal injection, 3 mice from each group were sacrificed and tumor mass was measured. Each of the four groups was then divided into two groups again by with or without 25 Gy of local lung radiation (IR), yielding a total of 8 groups, NC + PBS + no IR group, NC + NSC + no IR group, NC + PBS + IR group, NC + NSC + IR group, SH + PBS + no IR group, SH + NSC + no IR group, SH + PBS + IR group and SH + NSC + IR group (*N* = 6). One week after IR, 3 mice from each group were sacrificed, tumor mass was measured and the contralateral (left) lung was collected for H&E staining. Four weeks after initial tumor-bearing, that is, 2 weeks after IR, no mice from the four no IR groups survived. Mice from the remaining four IR groups were sacrificed, tumor mass was measured and the contralateral (left) lung was collected for H&E staining.

### Cell lines

Mouse lung epithelial cell line, MLE-12 (ATCC® CRL-2110™), and mouse lung cancer cell line, LLC (ATCC® CRL-1642™), were purchased from ATCC. MLE-12 was cultured with DME/F12 1:1 (1×) medium and LLC with DMEM/HIGH GLUCOSE medium, and both medium were added with 10% FBS and 1% Antibiotic-Antimycotic. Cells were cultured in an incubator under a condition of 5% CO_2_, 37 °C.

### Construction of Rac1 knockdown cell lines

Rac1 knockdown and control lentiviral vectors were purchased from OBiO Technology (Shanghai) Corp., Ltd. A total of 3 targeting-sequences were designed for RAC1 gene (Gene ID: 19353): GGAGACGGAGCTGTTGGTAAA (shRNA1), ATGTCCGTGCAAAGTGGTATC (shRNA2), GCTTGATCTTAGGGATGATAA (shRNA3). The lentiviral vectors used were pleno-gph, and the element order was CMV-MCS-EF1α-GFP-t2a-PURO. The successful construction of lentiviral vector interfering RAC1 gene was proved by sequencing analysis. MLE-12 and LLC cells were seeded in 12-well plates in a density of 1 × 10^5^ cells per well. 500 μl of culture medium was added, Rac1-shNC (control virus) and 3 targets of Rac1-SH (Rac1 knockdown virus) were added with a MOI of 20 (for MLE-12) or 10 (for LLC), respectively. Polybrene was added with a final concentration of 1:1000. After 24 h of virus infection, 500 μl of culture medium was added and cells were incubated for another 24 h. Successful virus infection was confirmed by a fluorescence microscope. The blank group without virus infection was used as a control for drug screening with puromycin. The culture medium was changed again with puromycin added. The doses of puromycin used for MLE-12 and LLC were 5 μg/ml and 20 μg/ml, respectively. After 3 days of drug screening, all cells in the Blank group died and surviving cells from Rac1-shNC and Rac1-SH groups were collected for infection efficiency detection by flow cytometry and Rac1 knockdown detection by qPCR and Western Blotting (Supplemental Materials, S2). RAC1-RNAi (1) was proved to have the strongest effect and used for subsequent experiments. Rac1 knockdown and control MLE-12/LLC were written as Rac1-shNC and Rac1-SH MLE-12/LLC, respectively.

### Construction of Trp53inp1 overexpression cell lines

Trp53inp1 (Gene ID: 60599) overexpression and control lentiviral vectors were purchased from OBiO Technology (Shanghai) Corp., Ltd. The lentiviral vectors element order were pLenti-CMV-Trp53inp1-HA-PGK-blasticidin and pLenti-CMV-MCS-HA-PGK-blasticidin for overexpression and control, respectively. Trp53inp1 overexpression (OE) and negative control (NC) MLE-12 cell lines were constructed on the basis of Rac1-NC and Rac1-SH MLE-12 cells, yielding a total of 4 kinds of genetically modified MLE-12 cells, which were Rac1-NC+ Trp53inp1-NC (represented as NC-NC), Rac1-SH+ Trp53inp1-NC (represented as SH-NC), Rac1-NC+ Trp53inp1-OE (represented as NC-OE) and Rac1-SH-Trp53inp1-OE (represented as SH-OE). Rac1-shNC and Rac1-SH MLE-12 cells were seeded in 12-well plates in a density of 1 × 10^5^ cells per well. 500 μl of culture medium was added, Trp53inp1-NC (control virus) and Trp53inp1-OE (overexpression virus) were added with a MOI of 20. Polybrene was added with a final concentration of 1:1000. After 24 h of virus infection, 500 μl of culture medium was added and cells were incubated for another 24 h. The culture medium was changed again with blasticidin (Product # A1113903, Thermo Scientific™, 200 μg/ml) added.

### Apoptosis detection by flow cytometry

Annexin V-FITC/PI Cell Apoptosis Detection Kit (TransGen Biotech Corp., Ltd, Beijing, China) and Annexin V, 633 Apoptosis Detection Kit (Dojindo Corp., Ltd, Shanghai, China) were used for apoptosis analysis of wild-type cell lines and virus-infected cell lines, respectively. Specific operation methods were proceeded according to the manufacturer’s instructions. Cells were fully digested with trypsin (without EDTA) for 8 min, the original medium was used for terminating cell digestion. After centrifuging at 4 °C, 1000 rpm for 5 min, the supernatant was discarded, the precipitate was resuspended in PBS and centrifuged again, and the supernatant was discarded. Cells were resuspended in 100 μl 1 × Annexin V Binding Buffer. 5 μl of Annexin V and PI reagents were added, samples were incubated at room temperature and protected from light for 15 min. Apoptosis ratio was detected by flow cytometry instrument.

### EdU cell proliferation detection by flow cytometry

BeyoClick™ EdU-594 Cell Proliferation Kit with Alexa Fluor 594 was purchased from Beyotime Biotechnology. Cell preparation and treatment were done according to manufacturer’s instruction. Detection was done by flow cytometry instrument.

### Western blotting (WB)

Cells were plated in medium dishes with a density of 1 × 10^6^ cells per dish. After corresponding treatments, proteins were extracted at corresponding time points. Culture medium was discarded and cells were washed twice with PBS. Protease inhibitor and phosphatase inhibitor were added 1:100 into RIPA Lysis and Extraction Buffer (Product # 89900, Thermo Scientific™) for protein lysate preparation. 150 μl of lysate was added to each sample and cells were lysed on ice for 10 min. Lysate was scraped off and cells were fully lysed with an ultrasonic cell disruption instrument. Lysate was centrifuged at 12,000 rpm for 15 min at 4 °C, 150 μl of supernatant was acquired for each sample and 37.5 μl of 5 × Protein Sample Loading Buffer (Product # LT103, Epizyme Biotech, China) was added, the mixture was boiled at 100 °C for 10 min.

7.5% (Product # PG111) and 12.5% (Product # PG113) PAGE Gel Fast Preparation Kits were purchased from Epizyme Biotech Corp., Ltd, Shanghai, China. Gels were prepared according to the manufacturer’s instructions. Beta Tubulin Antibody (Catalog number: 66240-1-Ig), GAPDH Antibody (Catalog number: 60004-1-Ig), BAX Antibody (Catalog number: 50599-2-Ig), and BCL2 Antibody (Catalog number: 12789-1-AP) were purchased from Proteintech^TM^, Wuhan, China. Anti-Caspase-3 antibody (ab13847), Anti-Cyclin D1 antibody (ab134175), Anti-CDK1 antibody (ab131450), Anti-Cyclin B1 antibody (ab181593), Anti-ATR (phospho T1989) antibody (ab227851), Anti-Chk1 (phospho S345) antibody (ab47318) and Anti-gamma H2A.X (phospho S139) antibody (ab11174) were purchased from Abcam (Shanghai) Corp., Ltd, China. Anti-Rac1 monoclonal antibody (Catalog Number: ARC03) was purchased from Cytoskeleton, Inc. Anti-Active Rac1-GTP Mouse Monoclonal Antibody (Catalog Number: 26903) was purchased from NewEast Biosciences. Anti-rabbit IgG, HRP-linked Antibody (7074) and Anti-mouse IgG, HRP-linked Antibody (7076) were purchased from Cell Signaling Technology (Shanghai) Corp., Ltd, China. Analysis of WB results was done by Image J software.

### Co-immunoprecipitation

Co-immunoprecipitation was conducted strictly in accordance with the manufacturer’s instruction provided by Cell Signaling Technology (Shanghai) Corp., Ltd, China. All operations were performed on ice in the following order: ① Cells cultured in 10-cm-dishes were washed with 1 ml of 4 °C pre-cooled PBS twice. ② 300 μl of pre-cooled cell lysate were added per dish and placed on ice for 5 min. ③ Cells were scraped off and transferred into a 1.5 ml centrifuge tube and fully lysed with an ultrasonic cell disruption instrument. ④ Lysate was centrifuged for 10 min at 14,000×*g*, 4 °C. ⑤ Supernatant was transferred into a new tube and added with 20 μg of Protein A and then placed in a 4 °C rotator for a full mixture for 1 h. ⑥ Mixture was centrifuged for 5 min at 3500 rpm, 4 °C, and the supernatant was transferred into a new tube, added with 2 μl of primary antibody, and put on the rotator for full mixture at 4 °C overnight. ⑦ 20 μl of Protein A was added on the next day and put on the rotator for mixture at 4 °C for another 4 h. ⑧After centrifuging at 3500 rpm, 4 °C, the supernatant was discarded and the pellet was resuspended with 500 μl of cell lysate and then centrifuged at 3500 rpm, 4 °C. This was repeated four 4 times. ⑨ Supernatant was discarded and 30 μl of 3 × protein loading buffer was added. ⑩Samples were boiled in a 100 °C water bath for 5 min and stored at −20 °C.

### RNA-Seq analysis

Rac1-shNC and Rac1-SH MLE-12 were seeded in dishes of 60 mm diameter at a density of 1 × 10^6^ cells per dish. Each kind of cell was seeded in a total of 3 dishes and incubated for 24hs. Medium was removed and cells were washed with sterilized PBS for once and then lysated with 1 ml/dish TRIzol™ Reagent (Thermo Fisher Scientific (China) Corp., Ltd). The cell lysate was kept frozen with dry ice and sent to Shanghai OE Biotech Corp., Ltd for RNA-Seq analysis. Compared with Rac1-shNC (control) MLE-12, there were a total of 262 deferentially expressed genes in the Rac1-SH MLE-12, 205 of which were downregulated and 57 upregulated (*P* < 0.05, |log 2FC| > 0.58).

### Statistical analysis

Statistical analysis was conducted using SPSS19.0 software. Data were expressed as mean ± SEM. All experiments were repeated three times. Student’s *t*-test was used for comparing data between two groups. Analysis of Variance (ANOVA) was used when there were multiple groups and the SNK-q test was used for further multiple comparisons between groups. The difference was considered as statistically significant when *P* < 0.05.

## Supplementary information

Supplementary figures

## Data Availability

All data generated or analyzed during this study were included in this published article and its supplemental material.
