# Supplementary material for: Inhibition of Rac1 attenuates radiation-induced lung injury while suppresses lung tumor in mice
Source: Cell Death Discov. 2022 Jan 14;8:26. doi: 10.1038/s41420-021-00791-8 (PMC8760284; doi:10.1038/s41420-021-00791-8)
Supplement: Supplementary file 1 — Supplementary figures [file 41420_2021_791_MOESM1_ESM.pdf]

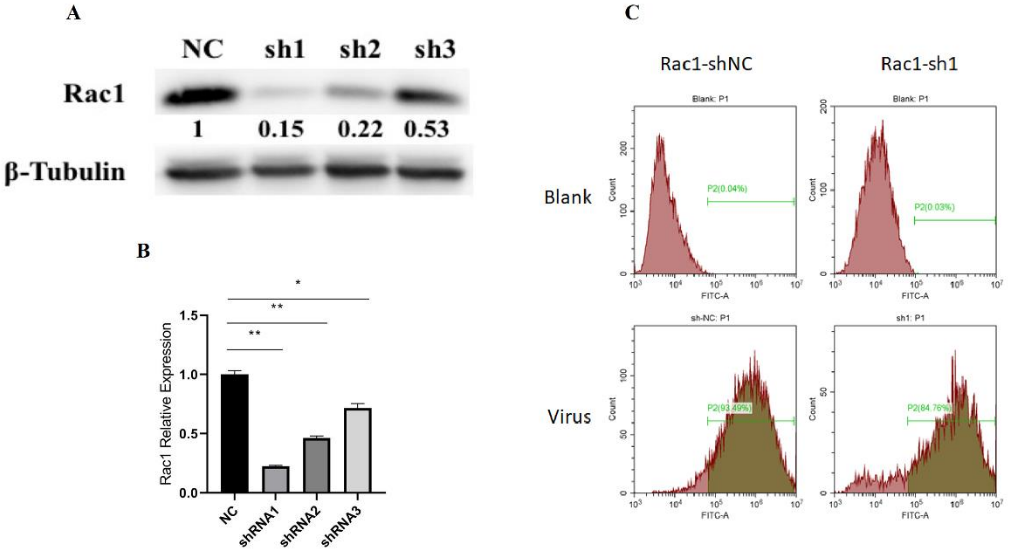

S1. Construction and efficiency validation of Rac1-knockdown MLE-12 cell lines. A total of 3 targeting-sequences were designed for RAC1 gene (Gene ID: 19353). (A) WB analysis of the expression level of Rac1. Compared with the control group (NC), all three shRNA lentiviruses could down-regulated the expression of Rac1, of which sh1 possessed the best efficiency. (B) qPCR analysis of the relative expression level of Rac1. (C) Virus infection efficiency detected by a fluorescence microscope. Blank groups were not infected with virus. The infection efficiency of Rac1-shNC (control) and Rac1-sh1 were 93.49% and 84.76%, respectively.

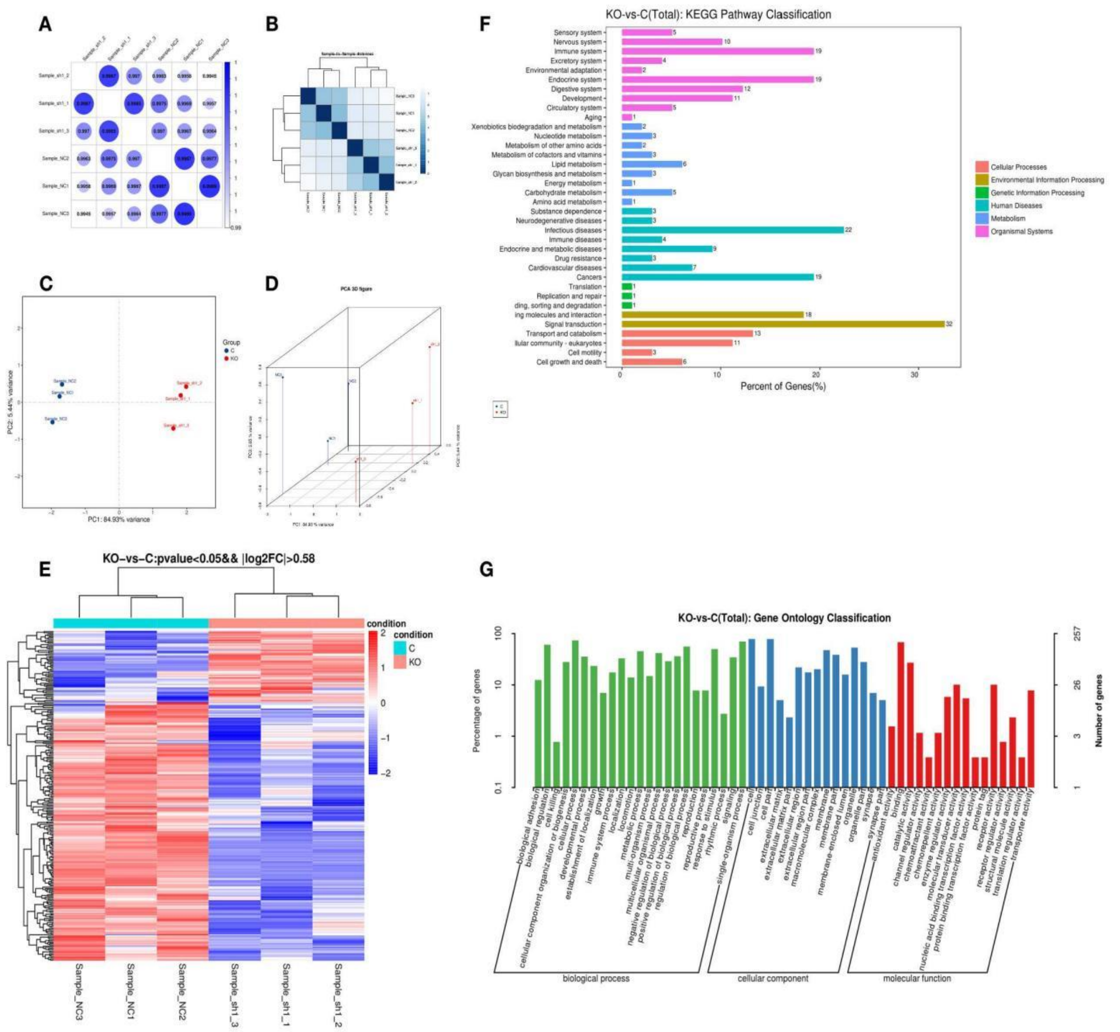

S2. Quality control of RNA-seq samples, Gene ontology and KEGG pathway analysis of differential expression genes caused by Rac1 knockdown. (A) Heat map of correlation coefficient between samples. (B) Sample-to-Sample cluster analysis results. (C) Principal component analysis (PCA analysis) of gene expression. (D) PCA analysis 3D graph. (E) Cluster analysis results of all differential expression genes. (F) Gene ontology (GO) analysis of differential expression genes caused by Rac1 knockdown. (G) KEGG pathway analysis of differential expression genes caused by Rac1 knockdown.

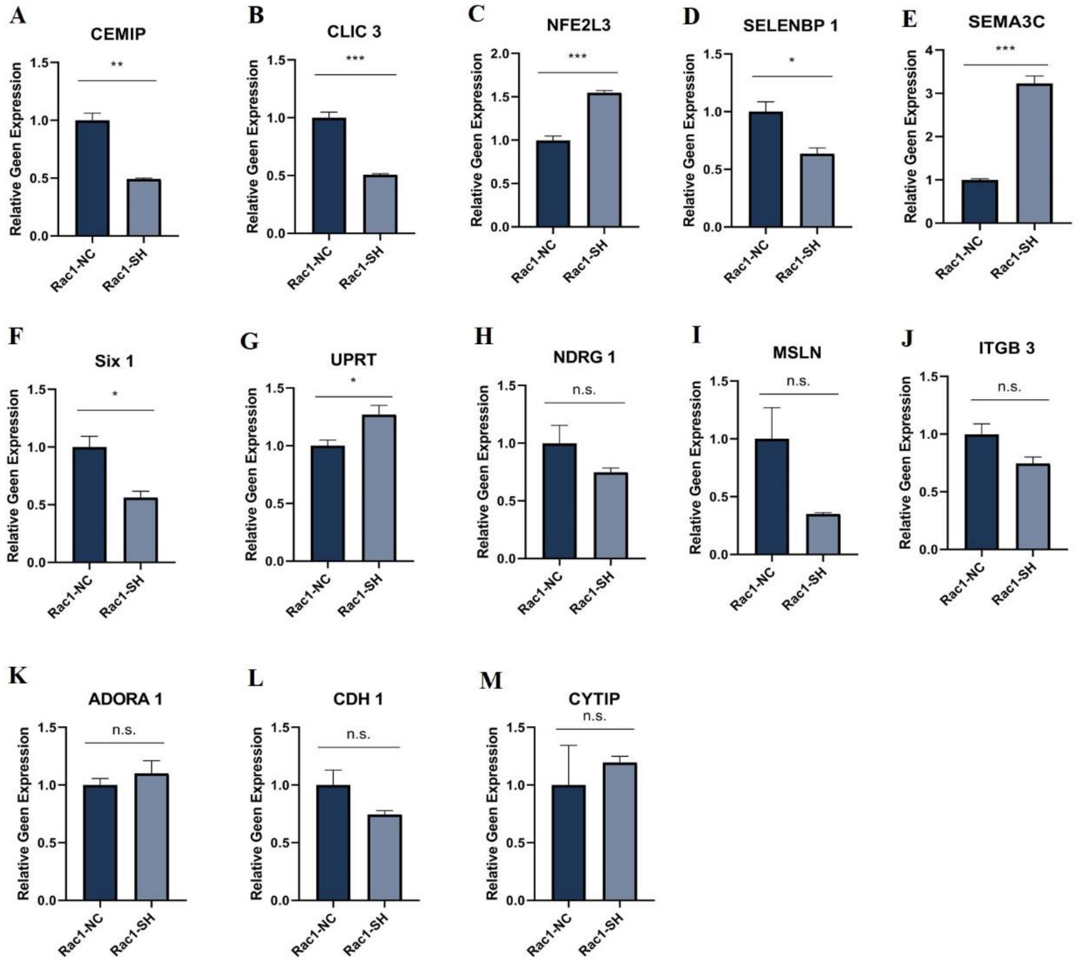

S3. qPCR analysis of another 13 differential expression genes caused by Rac1 knockdown.

A total of 14 differential expression genes (TRP53INP1 was shown in Figure 3F) were tested by qPCR. β-actin was used as the loading control. n.s. represented that there was no statistically significant difference between the two groups. *, ** and *** represented P <0.05, 0.01 and 0.001 between the two groups, respectively. The error value was expressed as mean±SEM, N=3.

A

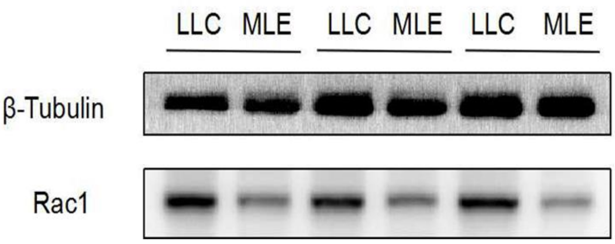

B

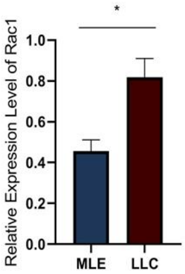

C

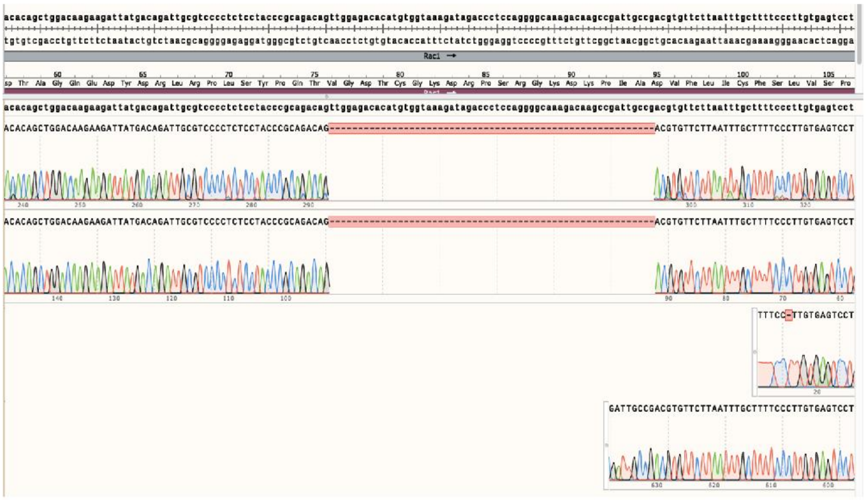

D

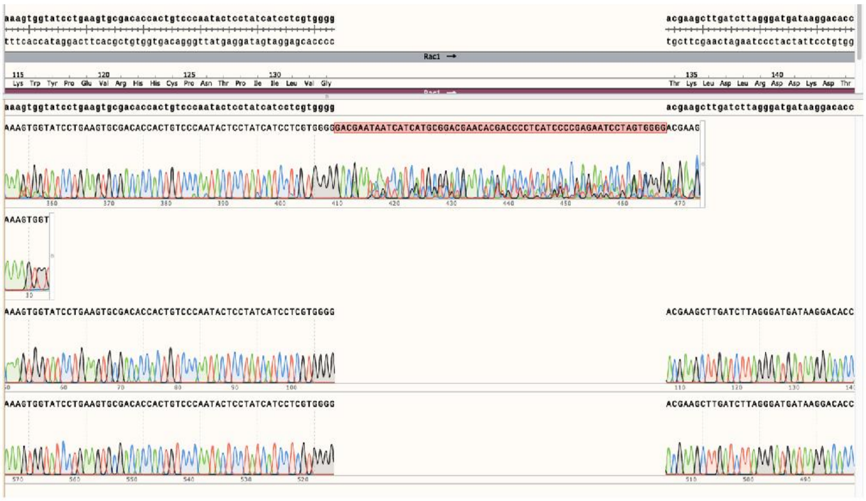

S4. The Over-expression and mutation of Rac1 between lung alveolar epithelial cell and lung

cancer cells (A) WB analysis of the Rac1 expression level in MLE-12 and LLC. (B) was the statistical analysis of the relative gray values of (A). (C-D) Exon sequencing of Rac1 in MLE-12 and LLC. Results showed that there were two insertion segments in the RAC1 gene of LLC, compared with that of MLE-12.Representive figures were displayed. *represented $P < 0.05$ between the corresponding groups, respectively. Experiment was repeated for three times.
